# Four heterozygous *de novo* variants in *ASXL3* identified with Bainbridge–Ropers syndrome and further dissecting published genotype–phenotype spectrum

**DOI:** 10.3389/fnins.2024.1456433

**Published:** 2024-11-14

**Authors:** Shengjie Ling, Yiming Zhang, Ning Li, Shan Tian, Rui Hu, Dongdong Zhang, Weitong Guo

**Affiliations:** ^1^Department of Reproductive Medicine, Central Hospital Affiliated to Shandong First Medical University, Jinan, China; ^2^Jinan Institute of Child Health Care, Children’s Hospital Affiliated to Shandong University, Jinan, China

**Keywords:** Bainbridge–Ropers syndrome, *ASXL3*, intellectual disabilities, nonsense variant, whole exome sequencing

## Abstract

Bainbridge–Ropers syndrome (BRPS) is a recently described neurodevelopmental genetic disorder associated with *de novo* truncating variants in additional sex combs like 3 (*ASXL3*) on chromosome 18q12.1. Trio-based exome sequencing was conducted on patients admitted to the Children’s Hospital Affiliated to Shandong University and diagnosed with unexplained intellectual disabilities or developmental delay between June 2022 and January 2024. *De novo* truncation of *ASXL3* was identified in four patients, and the pathogenic variants and their *de novo* status were validated using Sanger sequencing. Comprehensive clinical phenotype–genotype information of all previously reported patients with BRPS was collected and summarized. The common clinical manifestations observed in the four patients included language and intellectual disabilities or psychomotor retardation. Genetic analysis revealed that patient 1 carried a *de novo* heterozygous variant, c.1667_1668del (p.Thr556Arpfs*3), whereas patient 2 had a novel heterozygous frameshift variant of *ASXL3*, c.3324del (p.Lys1109Serfs*34). These two variants have not been documented to date. Additionally, patients 3 and 4 exhibited a *de novo* variant, c.4678C > T (p.Arg1560Ter). Based on the combined assessment of clinical phenotypes and genetic testing results, it was postulated that all four children presented with BRPS syndrome caused by pathogenic variations in *ASXL3*. The present study complements the range of *ASXL3* mutational and phenotypic spectra in the population, highlighting subtle distinctions in clinical manifestations between Chinese patients and other racial groups. The reporting of additional cases will contribute to further elucidating the function of *ASXL3* and establishing a solid foundation for clinical diagnosis and treatment.

## 1 Introduction

Bainbridge–Ropers syndrome (BRPS; MIM# 615485) is a neurodevelopmental disorder characterized by delayed psychomotor development, moderate to severe learning difficulties, behavioral problems, characteristic craniofacial features, hypotonia, and feeding problems. Characteristic facial changes include a prominent forehead, down slanting palpebral fissures, high-arched palate, full (everted) lower lip, downturned corners of the mouth, arched eyebrows, low columella, broad nasal tip, and anteverted nares. In 2013, Bainbridge et al. first identified *de novo* truncation variants in *ASXL3* that caused BRPS in four unrelated patients with similar phenotypes, using whole exome sequencing (Bainbridge et al., 2013). The estimated prevalence of *de novo* variants of *ASXL3* is approximately 1/193 (50/9625), positioning *ASXL3* among the top 10 genes associated with neurodevelopmental disorders in terms of *de novo* variant frequency (Wright et al., 2015).

*ASXL1* [MIM:612990], *ASXL2* [MIM: 612991], and *ASXL3* [MIM:615115], human homologs of the Drosophila additional sex comb (ASX) gene, serve as epigenetic scaffold proteins involved in the pathogenesis of non-cancerous diseases and cancers (Katoh, 2015). The *ASXL* family of genes shares domain architecture and biological functions and has been implicated in human diseases. *ASXL3* plays a functional role in deubiquitination and is expressed in several organ systems, including the central nervous system. Srivastava et al. showed that transcriptome analysis revealed >500 genes were differentially expressed in *ASXL3* patient fibroblasts relative to controls, and these genes were enriched for those involved in molecular processes impacting transcriptional regulation, proliferation, and development (Srivastava et al., 2016). Germline mutations in *ASXL1* and *ASXL2* are associated with specific genetic syndromes, such as Bohring–Opitz syndrome (BOS, OMIM #612990) (Fisher et al., 2003) and *ASXL2*-associated disorders (OMIM #612991) (Shashi et al., 2016). The overlap of phenotypes among these three diseases increases the difficulty of diagnosis and treatment. The scientific literature has documented 124 cases of BRPS, among which 92 different *ASXL3* variant types have been identified. The occurrence of BRPS has been reported in only 17 Chinese patients.

In this study, we conducted a comprehensive clinical evaluation of four Chinese patients diagnosed with BRPS. We further elucidated the phenotypic spectrum associated with *ASXL3* variants, identifying significant and recurrent clinical correlations that will enhance the precision of clinical management for individuals harboring pathogenic variants of this gene and facilitate informed decision-making in prenatal settings.

## 2 Materials and methods

### 2.1 Participants

Exome sequencing was conducted on patients admitted to the Children’s Hospital Affiliated to Shandong University and diagnosed with unexplained intellectual disability or developmental delay between June 2022 and January 2024.

### 2.2 Exome sequencing and data analysis

Genomic DNA was extracted from patient blood samples using the QIAamp DNA Blood Midi Kit (QIAGEN, Germany) following the manufacturer’s instructions. The FreySeq® Clinical Exome Library Preparation and Hybridization Capture Kit (V2.0 + mt) and FreySeq® Newborn Screening Library Preparation and Hybridization Capture Kit (V1.0), developed by Fuzhou Frey Medical Laboratory Co., LTD, were utilized for hybridizing with the gDNA library to enrich target DNA fragments, thereby constructing the target libraries. After passing quality control assessment, high-throughput sequencing was performed on an Illumina NovaSeq 6,000 sequencer (PE150), ensuring a minimum of 96% coverage at a depth of 20x.

Raw data underwent quality control procedures to generate clean reads for subsequent analysis. The clean reads were aligned to the human reference genome (GRCh37/hg19) using Sentieon software in order to identify SNPs and indels. Detected variants were filtered based on sequencing depth and mutation quality criteria to obtain high-confidence variant data. Variant data was annotated with population frequencies (e.g., gnomAD, 1000G, ExAC), pathogenicity databases (e.g., OMIM, HGMD, ClinVar), and functional damage predictions (e.g., Provean, SIFT, Polyphen2-HVAR, Polyphen2-HDIV, M-Cap, Revel, MutationTaster). Pathogenicity assessment was conducted according to the American College of Medical Genetics and Genomics (ACMG) guidelines (Richards et al., 2015). Sanger sequencing of *ASXL3* was conducted on whole-blood genomic DNA from patients and their parents to validate the pathogenic variants and their *de novo* status.

### 2.3 Ethics statement

Written informed consent for publishing clinical information was obtained from all legal representatives. The patients or their legal representatives provided written consent for the use of the images. This study was approved by the Ethics Committee of the Children’s Hospital Affiliated to Shandong University (SDFE-IRB/T-2024046).

### 2.4 Literature review

The PubMed and China national knowledge infrastructure databases were systematically searched using the search terms “Bainbridge Ropers” or “*ASXL3*” up to April 16, 2024. Duplicate and corrigendum articles were excluded and the remaining articles underwent relevance screening. A total of 128 probands with apparently heterozygous *ASXL3* variants were identified in the literature, including this study.

## 3 Results

*De novo* truncation variants in *ASXL3* were identified in four patients. The associated chromosomal aberrations in these patients had been excluded by chromosomal microarray analysis.

### 3.1 Clinical phenotype and *ASXL3* variant type

**Figure 1:**
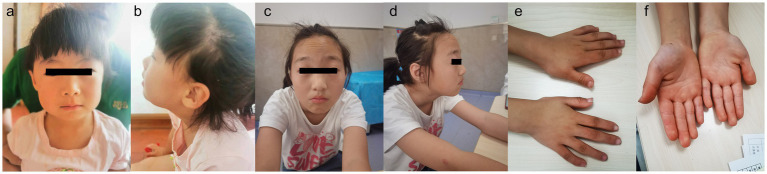
Patient 1, showing no any evident positive craniofacial features **(a,b)**. Patient 2, showing prominent forehead, arched eyebrows and downturned corners of the mouth **(c,d)**. Patient 2, showing bent fingers with slight ulnar deviation of the hand **(e,f)**.

Patient 1 is a 3-year-old female, the second offspring of a non-consanguineous Chinese couple. The patient’s five-year-old sister is healthy. The patient’s mother was pregnant at the age of 25 years. Her mother denied exposure to poisons, chemicals, or radiation during pregnancy or during regular prenatal examinations. The girl was born naturally at the 39th week of gestation, with a birth weight of 3.55 kg and length of 50 cm (Table 1). She achieved head control for 6 months and independent sitting for 11 months. She achieved unsupported standing at 13 months and unassisted walking at 20 months. The patient exhibits delayed language development and is currently limited to uttering the words “mom” and “dad” exclusively. The patient demonstrated responsiveness to names, maintained eye contact, responded appropriately to parental teasing, and denied any stereotypical behavior. The craniofacial features were unremarkable (Figure 1). Craniocerebral magnetic resonance imaging (MRI) revealed a partially enlarged exencephalic spaces.

**Table 1:** Summary of clinical data of the four patients and all reported individuals.

	Patient 1	Patient 2	Patient 3	Patient 4	Literature (124) + this cohort (total *n* = 128)
Gestation	C-section	Normal	IVF-ET	Intrauterine growth retardation	
Age	3y2m	12y	3y9m	2y11m	
Gender	F	F	M	M	
Birth weight	3.55	3.05	3.72	2.75	
Height	50	49	50	48	
variant	c.1667_1668del p.(Thr556Arpfs*3)	c.3382del (p.Lys1109Serfs*34)	c.4678C > T (p.Arg1560Ter)	c.4678C > T (p.Arg1560Ter)	92
Source	*De novo*	*De novo*	*De novo*	*De novo*	
ACMG	PVS1+PM2_Supporting +PS2	PVS1+PM2_Supporting +PS2	PVS1+PM2_Supporting +PS2	PVS1+PM2_Supporting +PS2	
Classification	Path	Path	Path	Path	
Gastrointestinal problem	−	+	−	+	91/112 (81.25%)
significant feeding problems	−	−	−	−	28/92 (30.4%)
Craniofacial features	−	+	+	+	
Downslanting palpebral	−	−	−	+	52/95 (54.7%)
High arch palate	−	−	−	+	46/93 (49.5%)
Prominent forehead	−	+	−	−	36/81 (44.4%)
Arched eyebrows	−	+	−	−	33/78 (42.3%)
Microcephaly	−	−	−	−	26/79 (32.9%)
Hypotonia	+	+	−	+	97/118 (82.2%)
Speech delay	delay	delay	non-verbal	non-verbal	114/114 (100%)
ID/DD	Mild	Mild	Severe	Severe	101/102 (99%)
Autism/ASD	No	No	Yes	No	32/81 (39.5%)
Seizures	No	No	No	No	33/116 (28.4%)
Brain abnormalities	+	−	−	+	22/83 (26.7%)

C-section, caesarian section; IVF-ET, in vitro fertilization-embryo transfer; ASD, autism spectrum disorder; F, female; M, male; ID, intellectual disability; DD, developmental delay; Path, pathogenic; ACMG criterion: PVS1: null variant (nonsense, frameshift, single or multiexon deletion) in a gene where loss-of-function is a known mechanism of disease, used at very strong level. PS2: De novo (both maternity and paternity confirmed) in a patient with the disease and no family history, used at moderate level. PM2_Supporting: Absent or had a low frequency of occurrence from controls in gnomAD database, used at moderate level.

A novel *de novo* heterozygous frameshift variant, c.1667_1668del (p.Thr556Arpfs*3), was identified in exon 11 of *ASXL3* and was predicted to result in a premature termination codon. This variant has not been previously reported. Sanger sequencing of family members validated this analysis, suggesting a *de novo* variant.

Patient 2 is a 12-year-old Chinese girl, born at term after an uncomplicated pregnancy. The patient’s mother was a primipara who became pregnant at the age of 22 years. Antenatal and perinatal history was normal. The girl was born at the 39th gestational week via cesarean section. At 12 months, she demonstrated the ability to vocalize “mom” and “dad” and began walking independently at 18 months. By the age of 3 years, her cognitive and comprehension abilities were observed to be comparatively delayed compared to those of typically developing children. Five years ago, the child exhibited inattentiveness, poor comprehension, severe learning difficulties, and academic underperformance. Currently, the child’s language expression abilities remain inadequate with unclear speech and word articulation. The child experienced feeding difficulties before the age of 2 years and frequently vomited during meals and feeding sessions. However, normal eating patterns were established after reaching 2 years of age. At the age of 12 years, growth parameters were: height 144.1 cm (10th percentile), weight 31.8 kg (10th percentile). Her facial features were characterized by arched eyebrows, a prominent forehead, and downturned corners of the mouth. The musculoskeletal features included bent fingers with slight ulnar deviation of the hand. Routine test results for complete blood count, liver and kidney functions, electrolytes, myocardial enzymes, and thyroid function were normal.

Therefore, trio-based whole-exome sequencing and subsequent validation using Sanger sequencing were performed. A novel nonsense variant in exon 12 of *ASXL3*, c.3382del (p.Lys1109Serfs*34), was also detected. Besides the primary variant, four secondary findings were revealed, including c.1805G > A (p.Arg602His) in *SMPD1* (NM_000543.4), c.11702C > A (p.Ser3901*) in *DYNC2H1* (NM_001080463.1), c.235del (p.Leu79Cysfs*3) in *GJB2* (NM_004004.5), and c.445_446del (p.Cys149Hisfs*32) in *MMACHC* (NM_015506.2). Secondary findings were excluded if they did not match the probands’ clinical features.

Patient 3 was referred for genetic evaluation at 3 years and 9 months of age. He was the only child of a healthy, non-biological couple. His mother used *in vitro* fertilization (IVF) to become pregnant at the age of 41 years and gave birth to the patient via cesarean section at full term. Developmentally, motor milestones were achieved appropriately; however, speech and language development were severely delayed. The boy acquired the ability to ambulate at the age of 14 months, but he currently presents with non-verbal communication skills. The individual does not respond when his name is called, avoids making eye contact with others, lacks initiative in greeting people, and shows little interest in playing with other children. He exhibited repetitive stereotypical behavior and was diagnosed with childhood autism. The patient had no feeding difficulties during the neonatal period. Results of cranial MRI, extensive metabolic testing, echocardiography, and creatine kinase level analysis were normal. Physical examination revealed a prominent forehead and widely spaced eyes.

Whole-exome sequencing revealed the *ASXL3* gene c.4678C > T (p.Arg1560Ter) variant (Table 1). The patient’s parents had no variants at this genetic locus, as confirmed by Sanger sequencing. Two secondary findings were revealed: c.2225C > T p.Thr742Met in *SMARCAL1* (NM_014140.3) and c.388G > Cp.Gly130Arg in *HGSNAT* (NM_152419.2). Secondary findings were excluded if they did not align with the proband’s clinical profile.

Patient 4 referred to our hospital is a 2-year and 11-month-old Chinese boy presenting with developmental delay. He was born to a nonconsanguineous Chinese couple. Antenatal visits revealed intrauterine growth retardation. The patient was born weighing 2.75 kg at full term (−2.1 SD) due to eutocia. The child exhibited delayed developmental milestones, achieving the ability to sit at 14 months and walk at 2.5 years. However, he still lacked language skills. The individual exhibited comprehension of basic oral communication, but encountered difficulties with complex instructions. Feeding difficulties occurred during the neonatal period but did not require tube feeding. The child did not exhibit any obvious stereotyped behavior or special interests. Facial features included a prominent forehead, down slanting palpebral fissures, and a high-arched palate. Regular laboratory tests, karyotyping, urine gas chromatographic mass spectrometry, and hearing screening were performed, and the results were negative. Electroencephalography findings were abnormal. Brain MRI revealed mild global white matter volume loss.

Finally, a heterozygous nonsense variant in exon 12, c.4678C > T (p.Arg1560Ter) of the *ASXL3* gene was detected. No variation was observed in the parents at this site. Sanger sequencing of the family members validated this analysis, suggesting a *de novo* variant.

Four patients diagnosed with *ASXL3* syndrome were selected from a population of approximately 674 individuals with intellectual disabilities or developmental delay. To date, 128 cases involving *ASXL3* variants have been documented, including 24 Chinese patients with *de novo* truncated *ASXL3* variants. Among them, there were 64 men and 62 women, ranging in age from 26 days to 47 years, except for one patient who underwent pregnancy termination at 33 weeks of gestation (Bacrot et al., 2018) and one patient who died aged 9 months (Bainbridge et al., 2013).

### 3.2 Clinical features in the literature

#### 3.2.1 Gestation

The age of the parents at the time of the patient’s birth was documented in 11 instances, with four cases involving both parents being over 35 years old and three cases where one parent was over 35 years old (Bainbridge et al., 2013; Chinen et al., 2018). Of the 75 patients for whom delivery mode information was available, cesarean section was performed in 38 cases. In terms of perinatal description, there were seven cases of intrauterine growth restriction, four cases of twin pregnancy, four cases of oligohydramnios, two cases of polyhydramnios, and eight cases of breech presentation. The prenatal histories of patients with BRPS may not reflect these characteristic features. However, it is important to consider certain factors, such as advanced gestational age, intrauterine growth restriction, and amniotic fluid abnormalities.

#### 3.2.2 Gastrointestinal problems and feeding difficulties

Feeding difficulty was observed in 81.25% of individuals. Among them, 28 patients presented with significant feeding problems, often accompanied by gastroesophageal reflux, necessitating interventions such as nasogastric tube feeding and fundoplication. The severity of feeding difficulties is most pronounced during infancy (Balasubramanian et al., 2017; Cuddapah et al., 2021; Schirwani et al., 2020). However, they gradually ameliorated as the children matured, leading to the resolution of symptoms related to reflux and vomiting.

#### 3.2.3 Cranio-facial features

Individuals with *ASXL3*-related syndromes have diverse facial characteristics. The most frequently reported craniofacial features in both domestic and foreign patients included down slanting palpebral fissures (52/95), high-arch palate (46/93), prominent forehead (36/81), arched eyebrows (33/78), and microcephaly (26/79). Chinese patients exhibit subtle facial characteristics, such as a prominent forehead and arched eyebrows. Additionally, the presence of facial features, such as a broad nasal tip, anteverted nostrils, a full (everted) lower lip, and an open-mouth appearance, has also been documented (Balasubramanian et al., 2017; Kuechler et al., 2017; Yu et al., 2021).

#### 3.2.4 Hypotonia

Gross motor skill development is frequently delayed in individuals diagnosed with *ASXL3*-related syndromes. The prevalence of hypotonia in individuals with *ASXL3*-related syndromes was 82.2% (of 97/118 cases). Hypotonia was more prominent during the neonatal and infancy periods. Gross motor skills are generally impaired in individuals with *ASXL3*-related syndromes. The average age of independent ambulation reported in the literature was 3 years and 7 months, with a subset of eight patients exhibiting the persistent inability to walk beyond the age of 4 years (Balasubramanian et al., 2017; Schirwani et al., 2021; Schirwani et al., 2023). However, among the seven Chinese patients who described their motor development, the average age at which they started walking was 20 months. It is worth noting that six of the patients with BRPS reported in China were under 12 months of age, which may explain this discrepancy.

#### 3.2.5 Speech delay and intellectual disability

**Figure 2:**
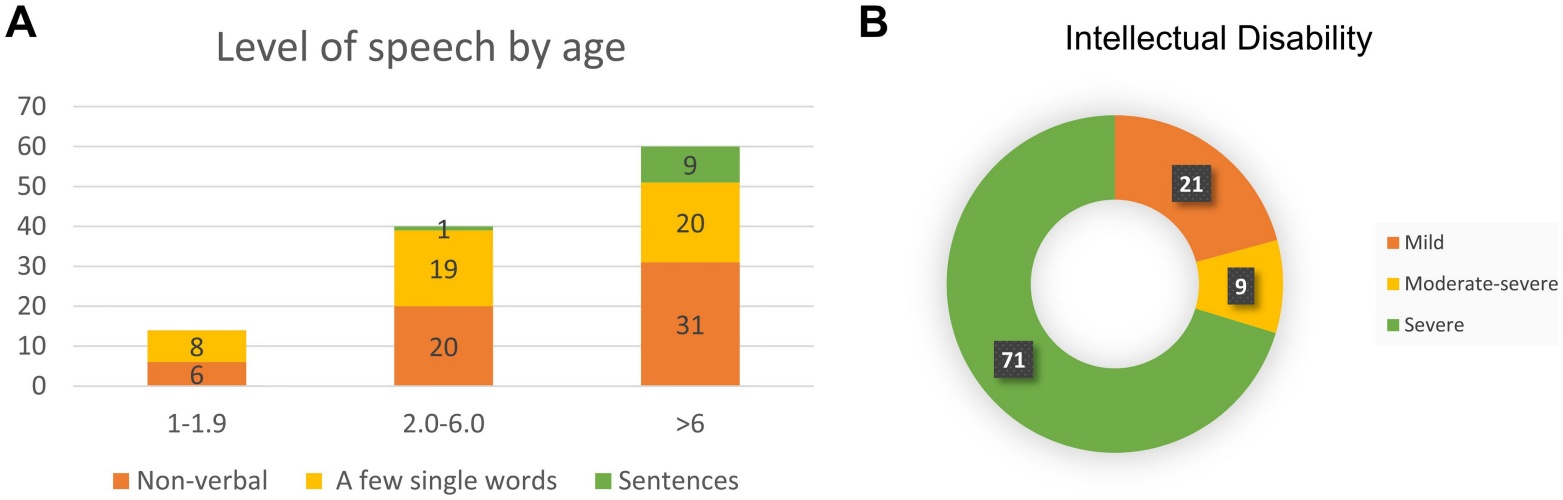
Bainbridge-Ropers syndrome individuals with language development **(A)** and intelligence assessment **(B)**.

All individuals with *ASXL3*-related syndromes exhibited significantly delayed speech and language development. In the literature, 49.1% of individuals exhibit complete nonverbal communication abilities. Approximately 42% demonstrated a limited vocabulary of fewer than 10 words, whereas a minority (8.9%) could express themselves in short sentences (Figure 2A). Language development retardation is closely associated with intellectual impairment, and the severity of intellectual disability can be evaluated (Figure 2B).

#### 3.2.6 Autism spectrum disorder

Patients diagnosed with or suspected of having autism spectrum disorder (ASD) accounted for 39.5% of individuals in the BRPS cohort (Table 1). Autistic traits, including stereotypies, poor eye contact, hand flapping, rocking, and head shaking, were present in most individuals. ASD is accompanied by coexisting conditions, such as developmental delay (DD), intellectual disability (ID), language disorders, motor difficulties, attention deficit hyperactivity disorder, and epilepsy (Hu et al., 2023). Hu et al. (2023) showed that, in a subgroup of children with ASD and DD, children with lower language ability have a greater probability of finding genetic variants. Nudel et al. (2021) considered pleiotropy between language impairment and ASD. Another hypothesis is that children with genetic conditions are more likely to display delays in early developmental milestones, especially language and motor functions. Therefore, genetic testing is necessary for children with ASD and DD or ID.

#### 3.2.7 Seizures and brain abnormalities

The 21 Chinese patients did not experience seizures, whereas 28.4% (33/116) of patients with BRPS from other racial backgrounds experienced seizures. The age of onset ranges from infancy to adolescence. However, cases of epilepsy with later onset occurring in adulthood have been documented (Khan et al., 2022). These findings indicate that the typical epilepsy phenotype in BRPS syndrome is childhood-onset generalized epilepsy with absence and generalized tonic–clonic seizures (Myers et al., 2018). The management of seizures generally exhibits a favorable response to standard antiepileptic drugs; however, achieving a complete cure remains challenging and necessitates lifelong medication (Schirwani et al., 2023). A more comprehensive understanding of the typical presentation and progression of epilepsy will contribute to accurate diagnosis, effective counseling, and informed treatment decisions. There were no characteristic findings on brain MRIs, and the majority of the 26.5% (22/83) who had abnormal brain MRI findings displayed white matter abnormalities, loss with enlarged lateral ventricles, and corpus callosum abnormalities.

### 3.3 Variants

**Figure 3:**
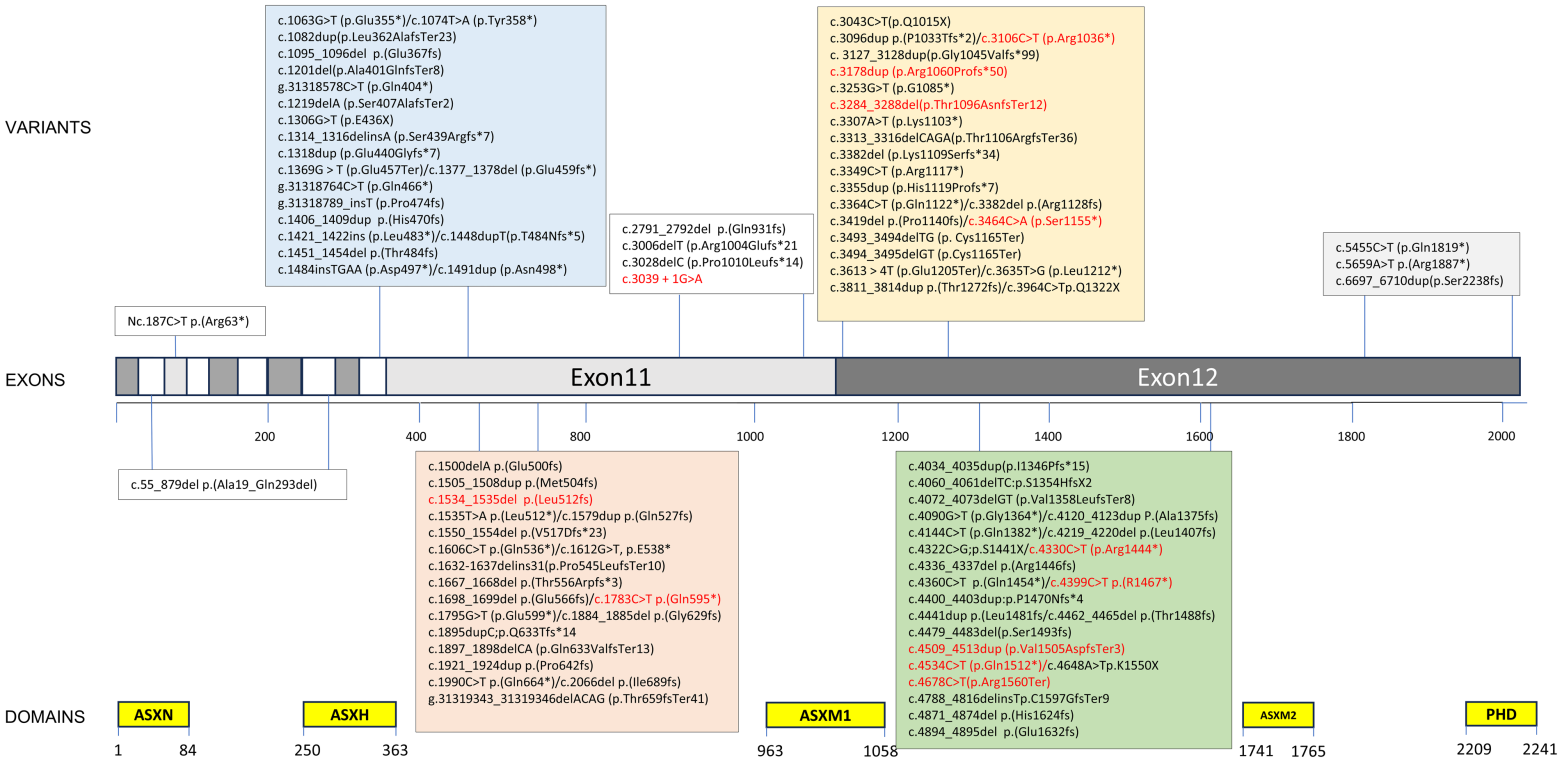
Variant spectrum of our cohort and previously published literature. ≥2 individuals marked in red. Variant nomenclature according to HGVS guidelines (http://varnomen.hgvs.org/) using National Center for Biotechnology Information (NCBI) reference transcript NM_030632.3.

**Figure 4:**
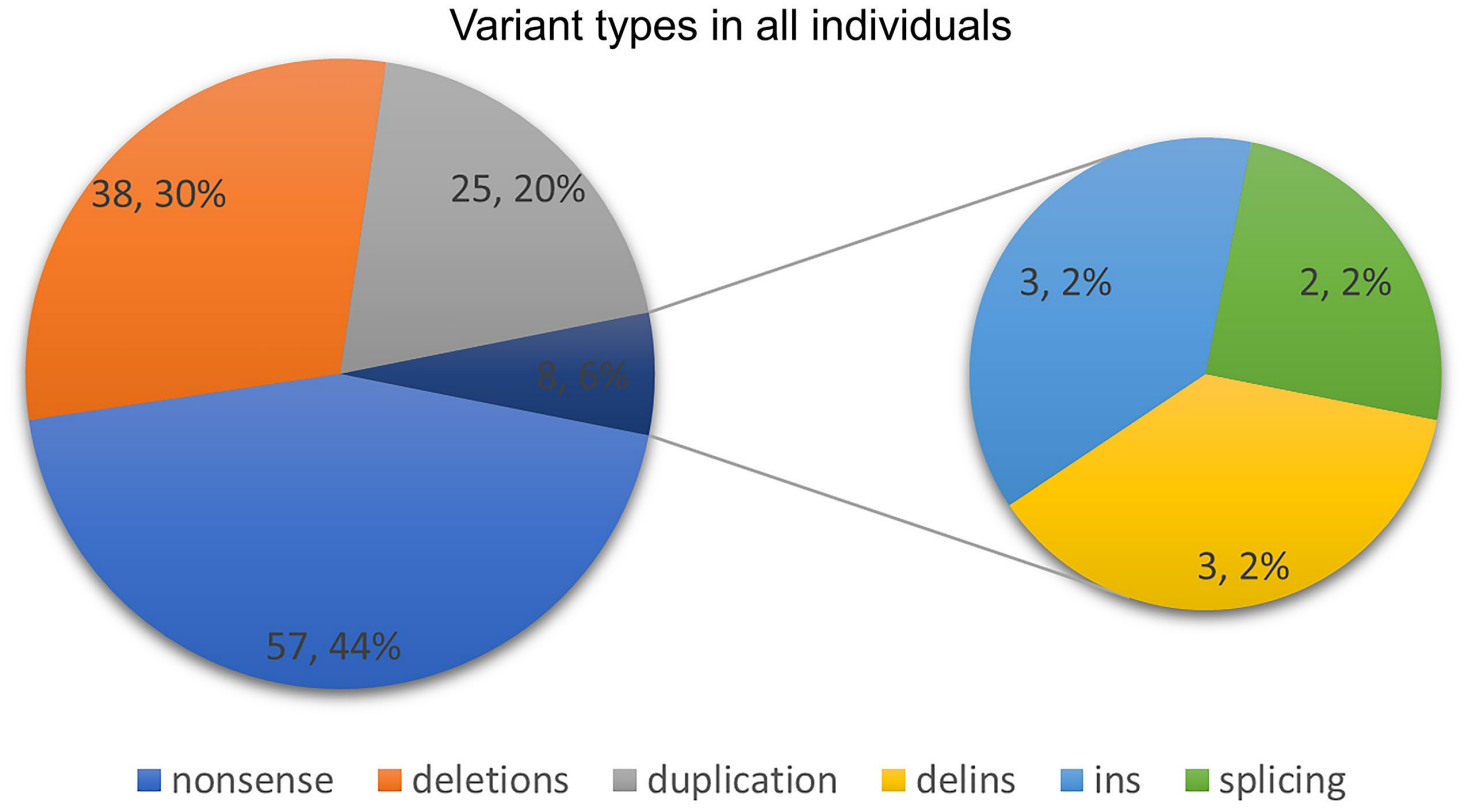
Variant types in our study and previously published literature.

Ninety-four *ASXL3* variants were detected in 128 patients. Of the variants identified in this study, 95.3% (122/128) were located in two exons (Figure 3). The majority of variants were *de novo* (Figure 4) and most cases were confirmed using parental genetic testing (Woods et al., 2024). Twelve variants were reported in more than one individual. The c.3106C > T p.(Arg1036*) (Schirwani et al., 2021; Myers et al., 2018; Aguilera et al., 2021; Koboldt et al., 2018; Duan et al., 2021; Gou et al., 2019) variant has been observed in eight individuals. Further analysis of the most frequent variant type revealed no significant correlation between the clinical phenotype and the mutation site, which aligns with previous findings (Balasubramanian et al., 2017). However, patients harboring the c.3106C > T (p.Arg1036*) variant exhibited consistent feeding difficulties, severe intellectual disabilities, and non-verbal communication. The c.4399C > T p.(Arg1467*) variant has been reported four times (Yu et al., 2021; Schirwani et al., 2021) and c.4330C > T p.(Arg1444*) has been reported five times (Srivastava et al., 2016; Balasubramanian et al., 2017; Schirwani et al., 2021), while c.4534C > T p.(Gln1512*) has been reported three times (Schirwani et al., 2023). The variation type c.4678C > T (p.Arg1560Ter) has been reported in three cases, including the two cases mentioned in this paper (Sumeyra Naralan et al., 2023). Two individuals were reported to have the remaining seven variants (marked in red in Figure 3).

## 4 Discussion

Additional sex combs-like 3 (*ASXL3*, MIM# 615115) was initially recognized in 2013 as a disease-causing gene based on a study involving four patients with *de novo* truncating pathogenic variants of this gene and a syndromal neurodevelopmental phenotype (Bainbridge et al., 2013). Bainbridge–Ropers syndrome has predominantly been reported in African American, Caucasian, Japanese, and Chinese populations (Yu et al., 2021). In this study, we identified four additional Chinese patients with BRPS. We have conducted a comprehensive analysis of 128 cases involving patients with detailed BRPS genotype–phenotype correlations, including 21 Chinese patients, and this review constitutes the most extensive case series to date.

Compared to other races, Chinese patients commonly exhibit BRPS characteristics such as atypical facial features, hypotonia, feeding difficulties, severe speech delay and intellectual disability, the absence of seizures, and behavioral phenotypes consisting of autistic traits. The clinical differences in individuals of different races with BRPS should be duly acknowledged in clinical practice, as this will facilitate the identification and diagnosis of the disease. Therefore, this is expected to require revision and expansion as additional patients have been documented with BRPS.

The availability of next-generation sequencing techniques has significantly facilitated the elucidation of genetic etiologies underlying well-characterized clinical syndromes, as well as those contributing to isolated intellectual disability (ID, IQ < 70) or newly described ID syndromes. Over 800 genes have been identified as causative for ID to date (Kochinke et al., 2016). *ASXL3* was identified using this methodology, and pathogenic variants of *ASXL3* are among the top 10 single-gene causes of developmental delay and intellectual disability (Wright et al., 2015). However, most studies on these disorders have focused on individuals of Northwest European ancestry (Deciphering Developmental Disorders Study, 2014), and the prevalence of *ASXL3*-related disorders has yet to be quantified. In this study, four of approximately 674 individuals with ID/DD were diagnosed with *ASXL3* syndrome (4/674). The frequency of *ASXL3* variants varies across cohorts and appears to depend on inclusion criteria (Kuechler et al., 2017).

*ASXL* genes (*ASXL1* at 20q11.21, *ASXL2* at 2p23.3, and *ASXL3* at 18q12.1) are orthologs of the Drosophila additional sex comb (Asx) gene, which encodes a regulator of the Polycomb-group repressor complex (PRC) (Katoh and Katoh, 2004). The conserved domains within the *ASXL* family include ASXN, ASXH, ASXM1, ASXM2, and the PHDs (Fisher et al., 2006). The *ASXL3* protein consists of 2,248 amino acids, with the majority of its gene coding sequence located in the final two exons, specifically exons 11 (1957 bp) and 12 (3,708 bp) of *ASXL3* (Katoh and Katoh, 2004). This characteristic accounts for the observed variations in the coding capacity within the *ASXL* gene family.

Most variants are *de novo* truncating variants with a predicted loss-of-function (Srivastava et al., 2016). The pathogenic mechanism is thought to involve a dominant-negative effect and functional haploinsufficiency (Bainbridge et al., 2013). The probability of loss-of-function intolerance (pLI) for *ASXL3* is reported to be 1, with an observed/expected (o/e) metric of 0.1, highlighting that the gene is highly intolerant to protein-truncating variants (Schirwani et al., 2021). This makes haploinsufficiency with high penetrance the most likely mechanism of BRPS (Kuechler et al., 2017). Haploinsufficiency may result from RNA-mediated decay, truncation, or complete loss of the *ASXL3* protein (Srivastava et al., 2016). Despite the identification of these variants, the exact functional role of *ASXL3* remains elusive. Ongoing investigations into *ASXL3* are crucial for enhancing both the scientific comprehension and clinical management of this condition.

The heterogeneity of this clinical condition is evidenced by the diversity in behavioral phenotypes and variations in intellectual development among siblings, with consistent genetic variants within families (Schirwani et al., 2023). Studies conducted by Ropers and Wienker (2015) and Fu et al. (2019) demonstrated the presence of incomplete penetrance in the genetic mutations observed in *ASXL3*. Therefore, more precise penetrance data are necessary to accurately estimate the disease recurrence rates. Determining the genetic status of the parents is essential for elucidating inheritance patterns and assessing potential recurrence risks in subsequent pregnancies. Even in instances where pathogenic variants are not identified in parental blood samples, a theoretical possibility of gonadal mosaicism persists, which may lead to the transmission of mutations to offspring (Wright et al., 2019). The identification of a pathogenic ASXL3 variant facilitates prenatal diagnosis (PND) and preimplantation genetic diagnosis (PGD), thereby empowering families to make informed decisions regarding future pregnancies.

Although we have conducted a comprehensive literature review of all previously documented cases, the sample size remains limited. Additionally, a multitude of variants have been documented with extremely limited clinical data, impeding efforts to delineate the phenotype associated with *ASXL3* related syndrome. Additional information from larger cohorts of individuals affected by BRPS is imperative in order to ascertain the complete phenotypic spectrum.

Our study presents four *de novo* variants in *ASXL3* that enrich the genetic spectrum and further emphasize the differences in clinical phenotypes between races with BRPS. The genotypic-phenotypic profiles of 128 cases of *ASXL3* syndrome identified to date were comprehensively summarized. Further investigations involving more case studies may be crucial to elucidate the function of *ASXL3*, thereby enhancing our understanding of the genetic etiology of this syndrome and facilitating accurate genetic counseling, informed decision-making, and prenatal diagnosis.

## Data Availability

The variation data reported in this paper have been deposited in the Genome Variation Map (GVM) in National Genomics Data Center, Beijing Institute of Genomics, Chinese Academy of Sciences and China National Center for Bioinformation, under accession number GVM000849.
